# Molecular Docking Studies and Biological Evaluation of Berberine–Benzothiazole Derivatives as an Anti-Influenza Agent via Blocking of Neuraminidase

**DOI:** 10.3390/ijms22052368

**Published:** 2021-02-27

**Authors:** Manu Kumar, Sang-Min Chung, Ganuskh Enkhtaivan, Rahul V. Patel, Han-Seung Shin, Bhupendra M. Mistry

**Affiliations:** 1 Department of Life Science, Dongguk University-Seoul, Ilsandong-gu, Goyang-si, Gyeonggi-do 10326, Korea; manukumar007@gmail.com (M.K.); smchung@dongguk.edu (S.-M.C.); 2 Department of Bio-resources and Food Science, Konkuk University, Seoul 143-701, Korea; enkhtaivan11@naver.com; 3 Department of Food Science and Biotechnology, Dongguk University-Seoul, Ilsandong-gu, Goyang-si, Gyeonggi-do 410-820, Korea; rahul.svnit11@gmail.com (R.V.P.); spartan@dongguk.edu (H.-S.S.)

**Keywords:** neuraminidase assay, antiviral activity, molecular docking, SRB assay

## Abstract

In this study, we have introduced newly synthesized substituted benzothiazole based berberine derivatives that have been analyzed for their in vitro and in silico biological properties. The activity towards various kinds of influenza virus strains by employing the cytopathic effect (CPE) and sulforhodamine B (SRB) assay. Several berberine–benzothiazole derivatives (BBDs), such as BBD1, BBD3, BBD4, BBD5, BBD7, and BBD11, demonstrated interesting anti-influenza virus activity on influenza A viruses (A/PR/8/34, A/Vic/3/75) and influenza B viral (B/Lee/40, and B/Maryland/1/59) strain, respectively. Furthermore, by testing neuraminidase activity (NA) with the neuraminidase assay kit, it was identified that BBD7 has potent neuraminidase activity. The molecular docking analysis further suggests that the BBD1–BBD14 compounds’ antiviral activity may be because of interaction with residues of NA, and the same as in oseltamivir.

## 1. Introduction

The extremely pathogenic influenza is probably the most commonly infected, severe respiration disease occurring seasonally in most countries. It remains a lethal disease due to the high rate of deaths caused by it. In most cases, high mortality rates in resource-limited nations are due to an insufficient supply of pharmaceutical drugs [1,2]. Influenza continues to be a severe health issue, and because of this infection, people of all ages consistently suffer. This pandemic respiratory disease might begin mainly by two essential mechanisms: transmission mechanism from birds to humans or genetic reassortment involving avian respiratory disease and human influenza viruses. Leading documented influenza were H7N9 (bird flu) of 2013, 2009 H1N1 (swine flu), 2005 H5N1 (bird flu), 1968 H3N2 (Hong Kong flu), 1957 H2N2 (Asian flu), and 1918 H1N1 (Spanish flu) [3,4,5,6]. Every year these epidemics are the leading causes of three to five million sicknesses and about 290,000 to 650,000 deaths worldwide [7].

Neuraminidase (NA), also referred to as sialidase, is the essential surface glycoprotein of the influenza virus that performs a vital and unique role in the influenza virus life cycle. It provides relief from virus progression. It is an essential enzyme in the infestation, maturity, replication, and distribution of the influenza virus [8,9,10]. There are currently two primary approaches available against the virus: small molecule anti-influenza drugs and vaccines. Oseltamivir and zanamivir are the two commercially potent NA inhibitors that cure influenza A and B infections [11,12]. 

Berberine, a plant-derived isoquinoline organic compound, having a protracted history of therapeutic use in Chinese drugs and Ayurveda. It belongs to Ranunculaceae, Papaveraceae, and Berberidaceae families. It is isolated from *Berberis vulgaris*, *Berberis petiolaris*, *Berberis aristata*, *Berberis asiatica*, *Berberis thunbergii*, *Berberis aquifolium*, *Coptis chinensis*, *Coptis teeta*, *Caulis mahoniae*, *Hydrastis canadensis*, and *Phellodendron amurense*. Berberine has significant actions towards antiviral activity [13,14,15,16], antidiarrheal effect [17], hypotensive effect [18], antibacterial effect [19], nuclear factor kappa-light-chain-enhancer of activated B (NF-κB) pathway [14], epidermal growth factor receptor (EGFR)/ mitogen-activated protein kinase kinase (MEK)/ extracellular-signal-regulated kinase (ERK) signal pathway [20], and AMPK/mTOR signal pathway [21]. 

Our study aims to produce a resistance-free antiviral drug. For that, we identified the potential effects of berberine–benzothiazole derivatives (BBDs) on the influenza virus and their active target site on the influenza virus by in vitro and in silico analysis. In this study, we examined BBDs for their anti-influenza action towards numerous strains of influenza virus employing CPE reduction screening with the SRB methodology. We found out that our newly synthesized BBDs exhibited a potent antiviral activity towards the influenza virus and collectively blocked viral NA activity. Furthermore, we have also applied a molecular docking study to evaluate the ligand–virus interactions of BBDs. To analyze the influence of binding ability of BBDs with the virus, the substituted BBDs, and the oseltamivir have been docked into the NA active site and their binding energies analyzed.

## 2. Results and Discussion

### 2.1. Cytotoxicity and Anti-Influenza Activity

Antiviral activity of the titled scaffolds BBD1–BBD14 was examined by applying an in vitro sulforhodamine B (SRB) bioassay. The final results were presented in Table 1. In the CPE method, various influenza viral strains (A/PR/34/8, A/Vic/3/75, B/Lee/40, and B/Maryland/1/59) were used with Madin-Darby Canine Kidney (MDCK) cells along with commercial drug oseltamivir as positive control drugs. The focus of the existing research was to improve the antiviral efficiency of berberine–benzothiazole components favorably through its well-rationalized derivatization, which is sufficient to discuss here that all title compounds revealed more significant antiviral results with half-maximal inhibitory concentrations (IC50s) varying from 24.28 ± 0.419–68.02 ± 0.670 µg/mL, 38.81 ± 2.51–70.65 ± 0.94, 36.94 ± 1.52–85.12 ± 3.74, and 36.88 ± 2.15–98.33 ± 2.21 towards influenza virus A/PR/34/8, A/Vic/3/75, B/Lee/40, and B/Maryland/1/59 strain. Antiviral analogs should not be cytotoxic towards the healthier cells; thus, the cytotoxicity levels of titled compounds towards MDCK cell lines are crucial. Presented CC50s in the variety 45.1 ±0.864–467.7 ± 2.647 µg/mL, which was throughout the bearable and therefore provided outstanding therapeutic indices (TI) of 4.835–19.08 (A/PR/34/8), 3.663–11.94 (A/Vic/3/75), 4.327–8.294 (B/Lee/40), and 3.851–7.924 (B/Maryland/1/59) respectively. Further, the existence of the electron-donating (ED) or electron-withdrawing (EWD) functional groups on the benzothiazole ring resulted in the molecules presenting a variable degree of antiviral effects. The most potent berberine–benzothiazole derivatives against the influenza virus A/PR/34/8 strain within the series tested was BBD7 with a chloro, benzothiazole functionalities attached to the berberine core analogue. Among all tested with 24.28 ± 0.419 µg/mL of IC50 and 463.5 ± 3.386 µg/mL of CC50 with most active TI of 19.08 when compared to its parent’s molecule berberine 36.12 ± 1.57 µg/mL of IC50 and 65.34 ± 1.92 µg/mL of CC50 and TI of 1.808. The oseltamivir control drug showed moderate activity (11.56 ± 1.43 of IC50, 205.3 ± 1.78 of CC50, and TI of 17.75). However, it can be noted that two other analogues appeared to have high antiviral efficacies as unsubstituted BBD1 and di-fluoro (BBD11) benzothiazole functionalities displayed 25.20 ± 0.154 µg/mL and 27.20 ± 0.394 of IC50 values and 17.32 and 17.19 of TIs, respectively. They had lower cytotoxic values of 436.7 ± 2.635 µg/mL and 467.7 ± 2.647 µg/mL, respectively.

With fact that the presence of a strong electron-withdrawing nitro group in BBD2 (IC50: 30.92 ± 0.619 µg/mL, CC50: 340.5 ± 1.180 µg/mL and TI: 11.01), BBD12 with trifluoromethyl group (IC50: 28.73 ± 0.514 µg/mL, CC50: 302.3 ±2.446 µg/mL and TI: 10.52), BBD10 with fluoro group (IC50: 30.50 ± 0.761 µg/mL, CC50: 310.6 ± 1.629 µg/mL and TI: 10.18), and acid group in BBD3 (IC50: 35.94 ± 0.659 µg/mL, CC50: 382.9 ±2.964 µg/mL and TI: 10.65), made up interesting efficacy against influenza virus A/PR/34/8 strain, respectively compared to those existing methyl (BBD4; IC50: 37.27 ± 0.669 µg/mL, CC50: 322.6 ± 2.543 µg/mL and TI: 8.655), methoxy (BBD5; IC50: 39.54 ± 0.326 µg/mL, CC50: 306.4 ± 3.612 µg/mL and TI: 7.749), ethoxy (BBD6; IC50: 42.77 ± 0.478 µg/mL, CC50: 221.4 ± 1.180 µg/mL and TI: 5.176), bromo group (BBD8; IC50: 68.02 ± 0.670 µg/mL, CC50: 328.9 ± 1.065 µg/mL and TI: 4.835), Iodo group (BBD9; IC50: 54.53 ± 1.750 µg/mL, CC50: 302.5 ± 1.771 µg/mL and TI: 5.547) and hydrazinobenzthaizole (BBD14; IC50: 40.34 ± 0.725 µg/mL, CC50: 304.3 ± 1.261µg/mL and TI: 7.543). All the compounds were more active than its parent berberine molecule in terms of TI values.

In the bioassay A/Vic/3/75 strain against derivative BBD7 having an EWD chlorine group demonstrated remarkable 38.81 ± 2.51 of IC50 with 11.94 of TI followed by an analogue BBD1 with 45.15 ± 1.75 of IC50 with 9.672 of TI and an analogue with 2,4-difluoro functionality in BBD11 with 48.44 ± 2.20 of IC50 with 9.655 of TI, respectively. These IC50 and TI values are far better than their parent berberine molecule, and TI values were similar to the control drug oseltamivir. Furthermore, in halogenated analogues, within the case of F and Cl having molecules (BBD7, BBD10, BBD11, and BBD12) observed higher antiviral effects than with Br and I containing molecules. Hence, forming the antiviral activity order of Cl > F > I > Br within halogenated groups. Among analogues, those carrying BBD3 with an acid group were found to have moderate antiviral activity, and ED groups like methyl, methoxy, and ethoxy functionality, also displayed moderated activity compared to control drug oseltamivir. However, when compared to berberine molecules, it showed potent activity in terms of TI values. A compound BBD2 with the nitro group and BBD14 without any substitution display a considerable level of antiviral action with 52.18 ± 2.07 of IC50 with 6.525 of TI, and 56.32 ± 2.06 of IC50 with 5.403 of TI, respectively, which is higher than the parental berberine molecule in terms of TI values. A compound BBD5 with an ED methoxy group attached to berberine core via alkyl chain was most active among all those studied against B/Lee/40 strain with 36.94 ± 1.52 of IC50 with 8.294 of TI, which was higher than berberine (60.83 ± 1.86 of IC50, 1.07 of TI) and control drug Oseltamivir (55.87 ± 1.13 of IC50, 3.67 of TI). Besides another unsubstituted analogue BBD1, BBD4 with ED methyl group and BBD3 with an acid group showed the highest level of B/Lee/40 strain inhibition potential comparable to that of control drug oseltamivir with 55.46 ± 3.77 of IC50 with 7.874 of TI, and 43.24 ± 0.86 of IC50 with 7.406 of TI, and 53.89 ± 1.36 of IC50 with 7.105 of TI, respectively. The data revealed that BBD5 (36.94 ± 1.52 of IC50, TI: 8.294) with methoxy group was more active than BBD6 (38.76 ± 2.08 of IC50, TI: 5.712) with an ethoxy group, which was closest to the control drug, oseltamivir, in value and was far better than the parent berberine molecule.

A compound EWD halo atom(s) demonstrated a remarkable structure–activity relationship as BBD7 with chlorine group having 81.42 ± 3.01 of IC50 with 7.105 of TI followed by BBD11 with di-fluoro (85.12 ± 3.74 of IC50 TI: 5.494), BBD10 with fluoro (64.64 ± 1.24 of IC50 TI: 4.812) and BBD12 with trifluoro functionalities (67.65 ± 1.52 of IC50 TI: 4.468), respectively. A compound BBD8 (74.82 ± 1.83 of IC50 TI: 4.395) with a Bromo group and BBD9 (69.84 ± 2.57 of IC50 TI: 4.331) with an iodo group were less active in halogenated groups. A compound BBD2 with the nitro group and BBD14 with an unsubstituted group displayed promising antiviral activity against B/Lee/40 strain. Overall, all the compounds exhibited more activity than the control drug oseltamivir and far better than parent berberine molecules.

Finally, an analogue without any substation BBD1 furnished a remarkable antiviral inhibitory efficacy level with 55.11 ± 2.65 of IC50 with 7.924 of TI against B/Maryland/1/59 strain, which was more active than control drug oseltamivir and much better than berberine. Furthermore, the functionality of compounds with ED groups of a BBD4-bearing methyl group, BBD5-bearing methoxy group, BBD6-bearing ethoxy group, had an appreciable level of antiviral activity against the B/Maryland/1/59 strain with 41.56 ± 0.67 of IC50 with 7.762 of TI, 44.27 ± 2.06 of IC50 with 6.921 of TI, and 36.88 ± 2.15 of IC50 with 6.003 of TI, respectively. Moreover, the presence of EWD halogenated groups such as chlorine (BBD7), fluorine (BBD10), difluoro (BBD11), trifluoromethane (BBD12), bromine (BBD8) and iodine (BBD9) groups connected to the berberine core and was effective for B/Maryland/1/59 strain with 88.13 ± 1.16 of IC50 with 5.259 of TI, 75.74 ± 2.06 of IC50 with 4.100 of TI, 98.33 ± 2.21 of IC50 with 4.75 of TI, 66.22 ± 1.82 of IC50 with 4.565 of TI, 81.16 ± 0.91 of IC50 with 4.052 of TI, and 78.55 ± 2.31 of IC50 with 3.851 of TI, respectively. 

The existence of two fluorine atoms enhanced the antiviral effect of BBD11 is in contrast with that of its mono-substituted fluorine atom BBD10. Furthermore, an analogue BBD2-bearing nitro group, BBD3-bearing acid group, and BBD14 without substitution had a remarkable antiviral activity against the B/Maryland/1/59 strain with 65.32 ± 1.88 of IC50 with 5.212 of TI, 56.44 ± 1.06 of IC50 with 6.784 of TI, and 73.44 ± 2.17 of IC50 with 4.143 of TI, respectively. Overall, the analogues showed active property against the B/Maryland/1/59 strain compared to the control drug oseltamivir and much better than the parent berberine molecule. The cyano group (BBD13) did not show any activity towards any influenza virus strains. Besides, previous research revealed that berberine and its derivatives confirmed strong anti-influenza activity via blocking of activity of influenza NA [14,15]. Therefore, we tested BBDs for inhibition of influenza NA activity via NA inhibition assay.

### 2.2. NA Inhibition Activity of BBDs

The NA of influenza virus is popularly known as sialidase. It consists of four similar subunits and attached to the membrane of the virus. NA, performs an essential role in the multiplication of the virus. The glycoproteins from neuraminic acid residues were recently found to have virion progeny type glycosidic linkage with the neuraminic acid receptor around the host-cell surface area; this glycosidic connection is divided by NA, which permits within the discharge from the virion progeny through the infected cells. Along these lines, NA is an alluring objective for anti-influenza research [22,23]. At present, three NA inhibitors have been widely used as anti-influenza drug-like zanamivir, oseltamivir, and peramivir. The protein action is also responsible for stopping the self-aggregation of virus particles by cleavage of sialic acids still certain towards the infection surface. We, subsequently, tried the potential activity of berberine–benzothiazole derivatives on the viral neuraminidase activity. Moreover, we tend to give many recently outfitted berberine derivatives that discovered potent anti-influenza activity via NA inhibition mechanism and examined by in vitro and in silico analysis. Notably, the past study indicated the role of berberine scaffolds mediated NA inhibition on the flu infection, which is determined by the NA inhibition assay and molecular docking measurements [14,15].

**Figure 1:**
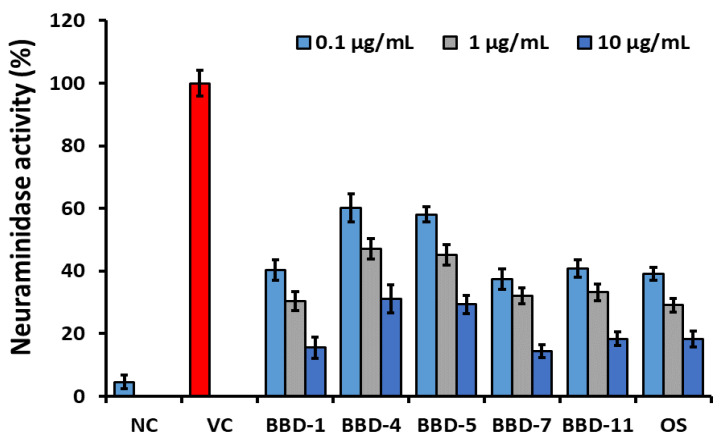
Neuraminidase activity of berberine derivatives comparing with oseltamivir. NC: Negative control. VC: Virus control. OS: Oseltamivir. The error bars are the mean of standard deviation in triplicate.

Furthermore, the NA inhibition assay was executed to determine the BBDs NA inhibition activity as shown in Figure 1. NA inhibition activity of BBDs was compared with standard NA inhibitor as oseltamivir. In the result, oseltamivir led between examined compounds along with NA activity was registered as 39.1% at 0.1 μg/mL, 29.1% at 1 μg/mL, and 18.3% at 10 μg/mL, respectively. On the other hand, NA activity among the analyzed BBDs, BBD7 was determined as the highest inhibition activity as dose-dependently on viral NA and are observed as 37.4% at 0.1 μg/mL, 32.1% at 1 μg/mL, and 14.5% at 10 μg/mL, respectively. An analogue BBD1 (40.3% at 0.1 μg/mL, 30.4% at 1 μg/mL, 15.6% at 10 μg/mL) and BBD11 (40.8% at 0.1 μg/mL, 33.2% at 1 μg/mL and 18.4% at 10 μg/mL) also displayed similar NA inhibition activity as oseltamivir. An analogues BBD4 and BBD5 showed moderate NA inhibition activity. This study’s outcomes indicated that the synthetic transform in the berberine molecule effectively provides alternative methods against influenza virus infections, and it can be applicable for another infectious respiratory disease such as COVID-19. 

### 2.3. Molecular Interaction between BBD and NA Protein

**Figure 2:**
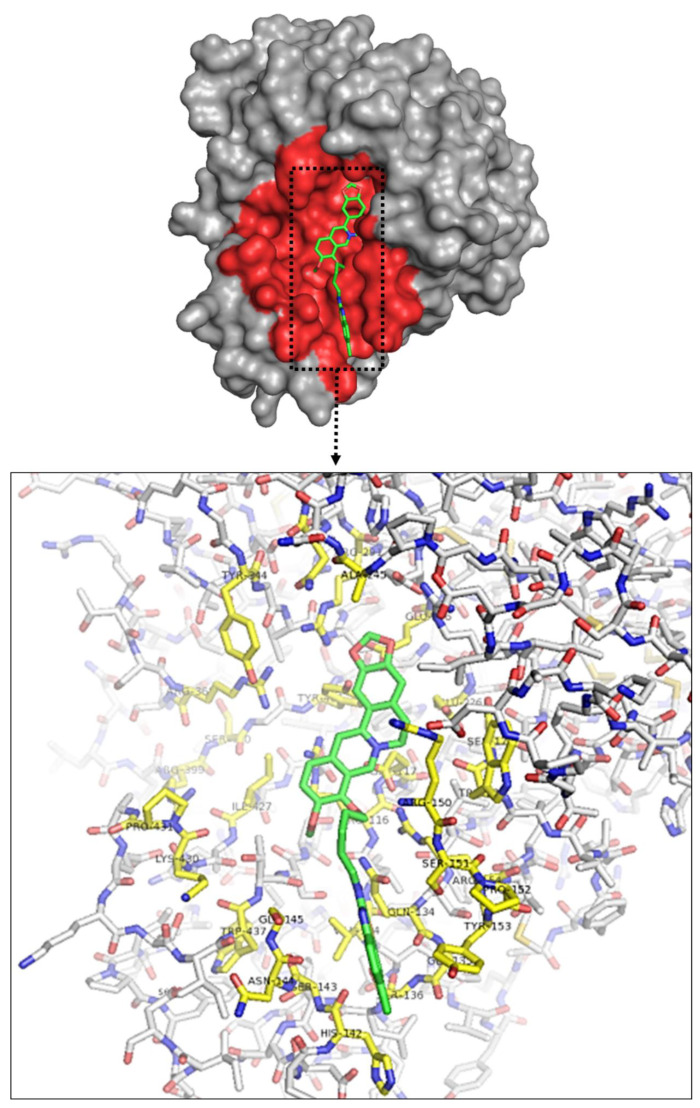
Crystal structure of influenza NA glycoprotein subunit docked with BBD-7. Pocked and important residues of influenza NA glycoprotein subunit colored as pink.

Protein–ligand interactions developed by molecular docking are the primary factor in identifying therapeutically essential structure-based enzyme inhibitor design [24]. To find out the interaction between BBDs and the active sites of NA, in silico molecular docking research was executed by using the AutoDock Vina program (Figure 2, Table 2, Table S1, PDB IDs: 4WA4).

**Figure 3:**
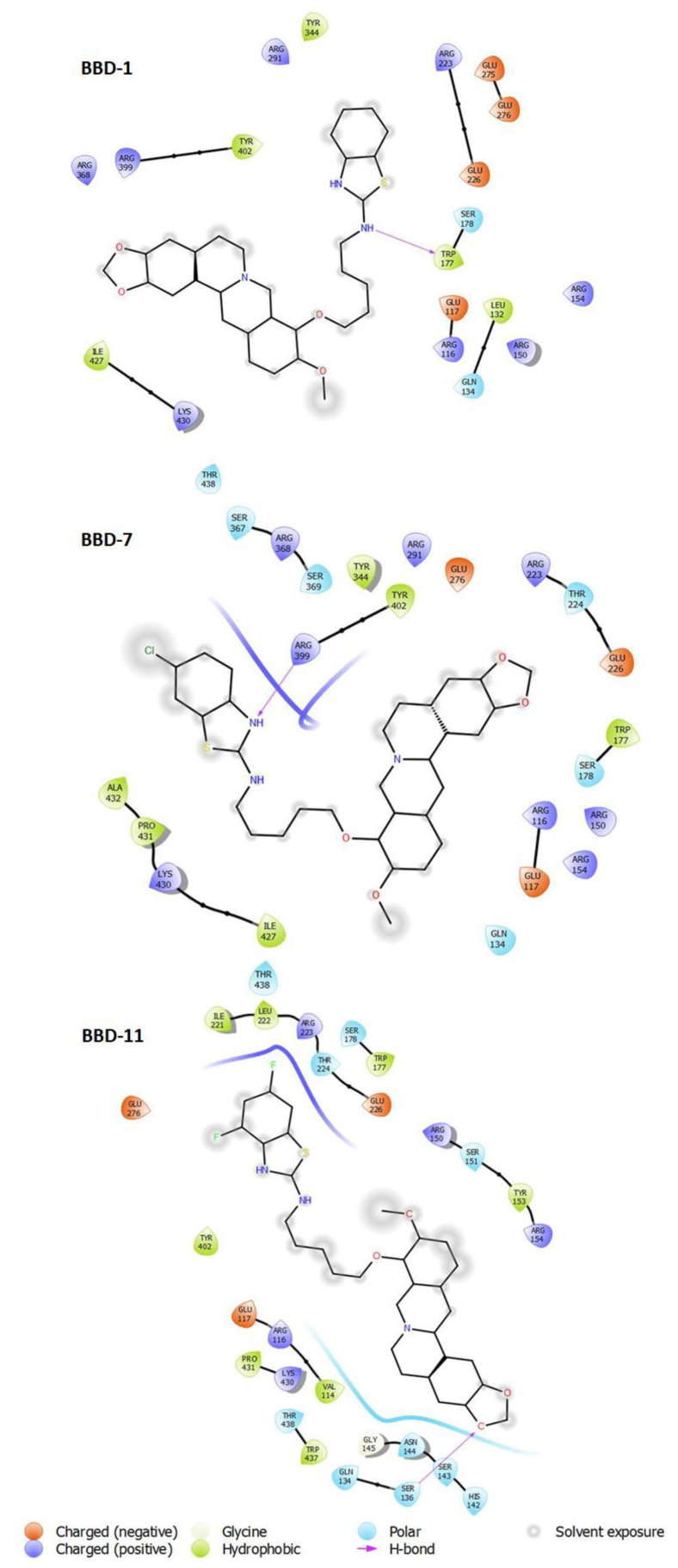
Overall binding interactions of BBD-1, BBD-7 and, BBD-11 were calculated in five Angstrom distances from the influenza NA subunit (4WA4). The ligand is represented in the best-scored pose.

There were nine best various poses and scores of BBDs discovered around the active site of influenza NA to compare with previously reported oseltamivir [18,19]. Among all the compounds, we conducted molecular docking of BBD1, BBD7, and BBD11 onto the NA crystal structure to know their neuraminidase inhibition. Figure 3 confirmed the docking poses of the BBD1, BBD7, and BBD11 ligands, and more significant binding activities were noticed as −7.9 kcal/mol, −8.4 kcal/mol, −8.0 kcal/mol, respectively, which is much better in comparison to oseltamivir (−6.1 kcal/mol). Besides, the ligand interaction between the BBDs and NA protein was determined by Maestro (Schrödinger, 2018) (Figure 3 and Table 3). 

The BBD1 contain amino acid residues as ARG291, TYR344, TYR402, ARG399, ARG368, ILE427, LYS430, THR438, ARG223, GLU275, GLU276, GLU226, SER178, TRP177, GLU117, ARG116, LEU132, GLN134, ARG150, ARG154, and BBD7 contain amino acid residues as SER367, ARG368, SER369, ARG399, TYR344, TYR402, ARG291, GLU276, ARG223, THR224, GLU226, TRP177, SER178, ARG150, ARG154, ARG116, GLU117, GLN134, ALA432, PRO431, LYS430, ILE427, THR438, and BBD11 contain amino acid residues as ILE221, LEU222, ARG223, THR224, SER178, TRP177, GLU226, ARG150, SER151, TYR153, ARG154, GLU276, TYR402, GLU117, ARG116, PRO431, LYS430, THR438, TRP437, GLN134, GLY145, ASN144, SER143, SER136, HIS142 while oseltamivir contains amino acid residues as ARG116, ARG150, ARG368, ARG291, GLU276 as previously reported [18,19].

Thus, we noticed that compounds BBD7 and BBD11 interact with active site residues, which suggest these ligands act selectively on the NA of the group and assist in tighter binding and improved activity.

## 3. Materials and Methods

### 3.1. Chemicals or Compounds 

The experimental procedure of synthesis of berberine–benzothiazole derivatives (BBDs) was previously reported [25]. Berberine received from Sigma-Aldrich Co., Ltd., Shanghai, China. Dimethyl sulfoxide (DMSO 0.1%, Sigma-Aldrich) was used to dissolve the berberine–benzothiazole and control compounds for the in vitro tests.

### 3.2. Reagents, Cells, and Viruses 

Madin–Darby Canine Kidney (MDCK) cells were carried out in Dulbecco’s Modified Eagle’s Medium (DMEM) supplemented with antibiotics (1% Penicillin, Gibco BRL, Grand Island, NY, USA) and 10% (*v*/*v*) fetal bovine serum (FBS). The cells were monitored at 37 °C in an incubator supplemented with 5% CO_2_. Tamiflu (Oseltamivir phosphate, Sigma) was beneficial for antiviral control. Influenza A viruses (A/PR/8/34 (H1N1, VR-1469), A/Vic/3/75 (H3N2, VR-822)), and influenza B viruses (B/Lee/40 (VR-1535), B/Maryland/1/59 (VR-296)) were acquired from the American Type Tissue Culture Collection (ATCC, Gaithersburg, MD, USA).

### 3.3. Cytotoxicity

An in vitro bioassay of antiviral activity of the berberine–benzothiazole (BBD1–BBD14) derivatives assessed by utilizing the sulforhodamine (SRB) technique to find out the cytopathic effect (CPE) affected by a viral infection, as recently reported [15,16]. Briefly, MDCK cells were cultured in a 96-well plate (1.5 × 10^4^/well) and monitored for the time being in a humidified cell culture incubator at 37 °C with 5% of CO_2_ supplement to allow attachment of the cells towards the wall of the 96-well plate. The final compounds’ stock solution was dissolved in DMSO and diluted with a DMEM medium to a suitable concentration. Then, phosphate buffer saline (PBS) was used to wash 96-well plates twice and then added final compounds at several concentrations (0.1, 1, 10, 100 μg/mL) to the plated in triplicate and incubated. The cells were fixed and washed, then recolored with SRB for 5 h after 48 h. The excess SRB stain was washed with 1% acetic acid, and the attached stain dissolved with tris-base [14,15]. The color intensity was measured by a SpectraMax Plus 348 microplate reader (Molecular Devices, USA) at 510 nm.

### 3.4. In vitro Antiviral Bioassay

A stock solution of influenza A virus (A/PR/8/34, A/Vic/3/75) influenza B virus (B/Lee/40, and B/Maryland/1/59) were diluted with DMEM medium containing trypsin–EDTA in serial dilutions followed by their 50% of tissue culture infective dose (TCID_50_) and which is utilized for virus infection. Briefly, MDCK cells were seeded in the 96-well plate (1.5 × 10^4^/well) overnight. The next day, the medium was removed and washed twice with PBS. Then, 90 µL of virus suspension (50 TCID_50_) and 10 µL medium having different concentrations of BBDs and oseltamivir solution (0.1, 1, 10, 100 μg/mL)) was added to 96-well plates for 48 h. All the treatments were maintained in triplicate for all concentrations, and the medium without samples was used as a control. The medium was removed after 48 h and washed twice with PBS. Then, 100 µL −20 °C 70% acetone was added. The 96-well plates were dried after removing acetone, and 100 μL of SRB (0.4 mg/L) was added. The excess of SRB was washed with a 1% acetic acid solution 4 to 5 times and dried again. AxioVision software (Carl Zeiss, Germany) was used to record cell images and also allotted to see the morphology of the cells, and once this observation, the SRB strain was dissolved with 100 μL of 10 mM of Tris base. A SpectraMax Plus 348 microplate instrument (Molecular Devices, USA) was used to record spectrophotometric data at 510 nm to analyze the cytotoxic concentration of 50% (CC_50_), inhibition concentration of 50% (IC_50_), and therapeutic indices (TI) [14,15].

### 3.5. Viral Neuraminidase Inhibition Assay

A standard fluorimetric assay was done to decide the impact of BBDs on the influenza virus neuraminidase activity (NA) by using the standard method with minor modification as reported [14,15]. NA inhibition activity was carried out by using NA-Star^®^ Influenza NA Inhibitor Resistance Detection Kit (Applied Biosystems, MA, USA). Briefly, NA assay was performed by making the reaction mixture containing an acetate buffer with influenza A/PR/8/34 virus strain, tested compounds (at concentrations 0.1, 1, 10, 100 µg/mL), and a 50 µL of NA-Star^®^ were incubated at 37 °C for 30 min at 5% of CO_2_. The reaction was started by adding 10 µL of NA-Star^®^ substrate and incubated at 37 °C for 30 min. The reaction was terminated by adding 60 µL of NA-Star^®^. The compounds’ fluorescence intensity was measured by using the SpectraMax L luminescent microplate reader (Molecular Device, CA, USA).

NA activity (%) = (Treatment/Virus) × 100.
Treatment: Virus + Compound
Virus: Virus

### 3.6. Molecular Docking Study

The AutoDock Vina program (Version 1.1.2, available at http://vina.scripps.edu accessed on 15 February 2021) used to perform in Silico molecular docking of BBDs towards influenza viral neuraminidase [26]. The Protein Data Bank (PDB, http://www.rcsb.org/pdb accessed on 15 February 2021) was used to find the crystal structures of the receptor (PDB IDs: 4WA4). The AutoDock-MGLTools (Version 1.5.6, http://mgltools.scripps.edu accessed on 15 February 2021) program was used for further receptor preparation [14,15]. The water molecules and heteroatoms were deleted from the protein. For the NA model, Kollman charges and all the polar hydrogen atoms were added and file as saved to a pdbqt file. BBDs and oseltamivir were docked after covering the catalytic site of NA with a grid box of 52(x) × 52(y) × 48(z), grid points being separated by 0.375 Å and centered at −2.6(x) × −5.06(y) × 13.6(z). The other parameters were carried out at their default settings. The outcomes were assessed by analyzing the ligand-protein interactions, the free energy of binding, and the RMSD values. All the docked structures of BPDs-enzyme complexes were imagined by applying for PyMol programs (Version 1.8.2, Schrodinger LLC). The 2D ligand interaction (5Å distance) were represented utilizing Maestro (Version 11.5.010, Schrodinger LLC).

## 4. Conclusions

In conclusion, a series of berberine–benzothiazole derivatives were discovered to be a new class of potential anti-influenza agents, and a total of 14 novel compounds were screened for the improvement of antiviral agents. The bio-assay results confirmed that the compounds BBD1–BBD14 exhibited exceptional antiviral activities towards influenza A and B virus strains, for example, A/PR/8/34, A/Vic/3/75, B/Lee/40, and B/Maryland/1/59 in cultured MDCK cells using oseltamivir as a controlled drug, among which compounds BBD1, BBD4, BBD5, BBD7, and BBD11 displayed outstanding antiviral activities. Furthermore, these compounds’ neuraminidase inhibitory activities (BBD1, BBD4, BBD5, BBD7, and BBD11) exhibited comparable NA activity than control oseltamivir drugs. Additionally, in silico research proposed that the compounds BBD1, BBD4, BBD5, BBD7, and BBD11 may create an inhibitory effect on the NA of influenza viruses because of attachment of ligand and NA active site residues and highest binding energy. Our results revealed that berberine–benzothiazole derivatives could show potent NA inhibitory activity. Its discovery could be utilized to develop novel influenza NA inhibitors.

## Figures and Tables

**Table 1:**
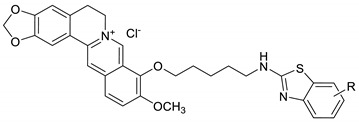
In vitro optimization of berberine–benzothiazole-based inhibitors.

BBD	“R” GROUP	MW	CC50 (µG/ML) ^a^	A/PR/8/34 (H1N1)		A/VIC/3/75 (H3N2)		B/LEE/40		B/MARYLAND/1/59		AFFINITY (KCAL/MOL)
				IC_50_ (µg/mL) ^b^	TI	IC_50_ (µg/mL) ^b^	TI	IC_50_ (µg/mL) ^b^	TI	IC_50_ (µg/mL) ^b^	TI	
**BBD 1**	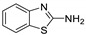	520	436.7 ± 2.635	25.20 ± 0.154	17.32	45.15 ± 1.75	9.672	55.46 ± 3.77	7.874	55.11 ± 2.65	7.924	−7.9
**BBD 2**	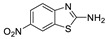	565	340.5 ± 1.180	30.92 ± 0.619	11.01	52.18 ± 2.07	6.525	61.72 ± 1.65	5.516	65.32 ± 1.88	5.212	−6.9
**BBD 3**	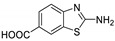	564	382.9 ± 2.964	35.94 ± 0.659	10.65	60.74 ± 1.06	6.303	53.89 ± 1.36	7.105	56.44 ± 1.06	6.784	−7.1
**BBD 4**	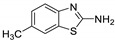	534	322.6 ± 2.543	37.27 ± 0.669	8.655	55.03 ± 0.87	5.862	43.24 ± 0.86	7.406	41.56 ± 0.67	7.762	−7.5
**BBD 5**	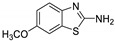	550	306.4 ± 3.612	39.54 ± 0.326	7.749	57.34 ± 2.13	5.343	36.94 ± 1.52	8.294	44.27 ± 2.06	6.921	−7.6
**BBD 6**	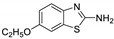	564	221.4 ± 1.180	42.77 ± 0.478	5.176	60.43 ± 1.35	3.663	38.76 ± 2.08	5.712	36.88 ± 2.15	6.003	−5.7
**BBD 7**	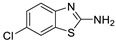	554	463.5 ± 3.386	24.28± 0.419	19.08	38.81 ± 2.51	11.94	81.42 ± 3.01	5.692	88.13 ± 1.16	5.259	−8.4
**BBD 8**	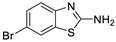	599	328.9 ± 1.065	68.02 ± 0.670	4.835	70.65 ± 0.94	4.65	74.82 ± 1.83	4.395	81.16 ± 0.91	4.052	−7.1
**BBD 9**	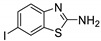	702	302.5 ± 1.771	54.53 ± 1.750	5.547	54.16 ± 2.09	5.585	69.84 ± 2.57	4.331	78.55 ± 2.31	3.851	−7.1
**BBD 10**	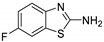	539	310.6 ± 1.629	30.50 ± 0.761	10.18	49.87 ± 1.31	6.228	64.64 ± 1.24	4.812	75.74 ± 2.06	4.100	−6.8
**BBD 11**	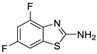	556	467.7 ± 2.647	27.20 ± 0.394	17.19	48.44 ± 2.20	9.655	85.12 ± 3.74	5.494	98.33 ± 2.21	4.75	−8.0
**BBD 12**	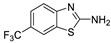	588	302.3 ± 2.446	28.73 ± 0.514	10.52	44.56 ± 1.65	6.784	67.65 ± 1.52	4.468	66.22 ± 1.82	4.565	−6.0
**BBD 13**	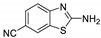	545	45.1 ± 0.864	ND	-	ND	-	ND	-	ND	-	−6.7
**BBD 14**	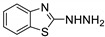	591	304.3 ± 1.261	40.34 ± 0.725	7.543	56.32 ± 2.06	5.403	70.32 ± 2.24	4.327	73.44 ± 2.17	4.143	−6.7
**BERBERINE**			65.34 ± 1.92	36.12 ± 1.57	1.808	41.58 ± 2.04	1.571	60.83 ± 1.86	1.07	54.63 ± 1.45	1.196	-
**OSELTAMIVIR**			205.3 ± 1.78	11.56 ± 1.43	17.75	22.14 ± 1.07	9.27	55.87 ± 1.13	3.67	44.17 ± 1.83	4.64	-

^a^ CC_50_: Concentration of the BBDs in μg/mL inhibiting 50% of virus-induced cytopathic effects. ^b^ IC_50_: The cytotoxic concentration of 50% on normal cells (MDCK) in μg/mL. ND; Not detected. The results are the mean of standard deviation (±S.D) in triplicate. TI: Therapeutic index (TI = CC_50_/IC_50_).

**Table 2:** Binding energies of the BBD-7 compared with oseltamivir on NA along with their Root Mean Square Distance value.

#	Affinity (kcal/mol) ^@^		RMSD L.B ^#^		RMSD U.B *	
	BBD-7	Ose	BBD-7	Ose	BBD-7	Ose
1	−8.4	−6.1	0	0	0	0
2	−8.1	−6.0	5.706	2.205	9.555	4.419
3	−8	−5.7	2.454	2.120	3.43	3.267
4	−7.9	−5.6	7.43	2.362	11.301	4.659
5	−7.6	−5.5	11.979	2.383	17.015	4.191
6	−7.6	−5.3	12.165	2.376	16.089	4.982
7	−7.5	−5.2	5.553	2.631	10.121	5.237
8	−7.4	−5.2	16.288	2.645	19.921	3.757
9	−7.4	−5.2	16.355	14.54	19.467	16.635

^@^ Binding energies between ligand and receptor (Affinity (kcal/mol)). ^#^ RMSD L.B: Distance from best mode root-mean-square deviation lower bound. * RMSD U.B: Distance from best mode root-mean-square deviation upper bound. Ose: Oseltamivir.

**Table 3:** The binding interaction residues registered between NA and compounds (distance five Angstroms).

Compounds	Residues
Oseltamivir	ARG116, ARG150, ARG368, ARG291, GLU276
BBD-1	ARG291, TYR344, TYR402, ARG399, ARG368, ILE427, LYS430, THR438, ARG223, GLU275, GLU276, GLU226, SER178, TRP177, GLU117, ARG116, LEU132, GLN134, ARG150, ARG154
BBD-7	SER367, ARG368, SER369, ARG399, TYR344, TYR402, ARG291, GLU276, ARG223, THR224, GLU226, TRP177, SER178, ARG150, ARG154, ARG116, GLU117, GLN134, ALA432, PRO431, LYS430, ILE427, THR438
BBD-11	ILE221, LEU222, ARG223, THR224, SER178, TRP177, GLU226, ARG150, SER151, TYR153, ARG154, GLU276, TYR402, GLU117, ARG116, PRO431, LYS430, THR438, TRP437, GLN134, GLY145, ASN144, SER143, SER136, HIS142

## Data Availability

Data is contained within the article or supplementary material.

## Supplementary Materials

The following are available online at https://www.mdpi.com/1422-0067/22/5/2368/s1.
