# Supplementary material for: Molecular Docking Studies and Biological Evaluation of Berberine–Benzothiazole Derivatives as an Anti-Influenza Agent via Blocking of Neuraminidase
Source: Int J Mol Sci. 2021 Feb 27;22(5):2368. doi: 10.3390/ijms22052368 (PMC7956587; doi:10.3390/ijms22052368)
Supplement: Supplementary file 1 [file ijms-22-02368-s001.pdf]

Table S1. Binding energies of the BBDs compared to NA along with their Root Mean Square Distance value.

BBD	Mode	Affinity (kcal/mol)	RMSD L.B#	Best mode rmsd u.b.
1	1	-7.9	0	0
	2	-7.7	5.585	8.371
	3	-7.7	9.92	13.661
	4	-7.5	10.793	13.24
	5	-7.5	6.739	10.132
	6	-7.4	7.381	10.224
	7	-7.4	27.02	30.878
	8	-7.4	9.996	13.486
	9	-7.2	5.951	8.61
2	1	-6.9	0	0
	2	-6.6	5.452	8.823
	3	-6.4	3.405	4.597
	4	-5.9	5.858	10.538
	5	-5.9	6.242	10.883
	6	-5.9	12.188	17.632
	7	-5.8	10.856	13.703
	8	-5.8	7.683	10.423
	9	-5.6	11.689	16.128
3	1	-7.1	0	0
	2	-7.3	2.278	4.097
	3	-6.6	2.865	6.147
	4	-6.3	5.397	6.681
	5	-6.3	9.079	13.012
	6	-6.3	2.904	5.434
	7	-6.2	8.618	12.661
	8	-6.2	4.36	8.627
	9	-6.2	10.503	14.444
4	1	-7.5	0	0
	2	-6.9	1.758	2.499
	3	-6.7	1.899	7.512
	4	-6.6	18.451	21.367
	5	-6.6	4.925	8.911
	6	-6.6	2.082	3.511
	7	-6.4	17.252	20.369
	8	-6.3	18.637	22.525
	9	-6.1	19.604	22.709
5	1	-7.6	0	0
	2	-6.9	4.181	6.503
	3	-6.3	5.197	9.824
	4	-6	9.399	13.438
	5	-6	5.309	9.043
	6	-6	16.618	21.03
	7	-6	16.823	20.51
	8	-5.9	19.392	22.539
	9	-5.9	10.717	14.705
	1	-5.7	0	0

	2	-5.6	4.084	8.709
	3	-5.5	15.191	18.768
	4	-5.5	5.504	10.015
6	5	-5.5	3.563	7.348
	6	-5.5	5.562	10.186
	7	-5.4	3.327	8.338
	8	-5.3	4.485	8.671
	9	-5.2	6.649	10.599
	1	-8.4	0	0
	2	-8.1	5.706	9.555
	3	-8	2.454	3.43
	4	-7.9	7.43	11.301
7	5	-7.6	11.979	17.015
	6	-7.6	12.165	16.089
	7	-7.5	5.553	10.121
	8	-7.4	16.288	19.921
	9	-7.4	16.355	19.467
	1	-7.1	0	0
	2	-6.6	9.704	12.72
	3	-6.3	10.773	14.759
	4	-6.3	19.82	23.875
8	5	-6.2	3.612	7.189
	6	-6.2	4.235	8.952
	7	-6.1	22.382	25.075
	8	-6.1	11.194	13.838
	9	-6.1	2.463	3.24
	1	-7.1	0	0
	2	-7	4.42	9.253
	3	-6.7	6.861	9.85
	4	-6.7	15.002	18.399
9	5	-6.5	8.84	13.67
	6	-6.5	3.835	8.004
	7	-6.4	3.835	8.972
	8	-6.4	23.17	26.479
	9	-6.3	10.106	13.097
	1	-6.8	0	0
	2	-6.7	3.41	6.765
	3	-6.6	2.515	4.63
	4	-6.6	5.452	7.822
10	5	-6.4	2.893	7.928
	6	-6.2	3.4	5.7
	7	-6.1	10.933	14.43
	8	-6	9.807	12.062
	9	-5.9	5.087	6.755
	1	-8	0	0
	2	-7.9	10.32	14.881
	3	-7.8	18.175	20.807
	4	-7.8	6.773	8.412
11	5	-7.7	13.102	16.012
	6	-7.7	7.685	14.034
	7	-7.5	7.284	9.964
	8	-7.5	3.927	5.074

	9	-7.5	6.449	9.089
12	1	-6	0	0
	2	-6	3.922	7.395
	3	-5.9	4.033	7.793
	4	-5.8	3.988	7.669
	5	-5.8	2.785	4.517
	6	-5.7	19.48	22.068
	7	-5.6	8.981	11.932
	8	-5.6	2.176	3.246
	9	-5.5	5.546	9.489
13	1	-6.7	0	0
	2	-6.6	3.721	8.632
	3	-6.2	6.567	9.524
	4	-6.1	4.951	7.255
	5	-6.1	2.261	3.612
	6	-6.1	18.45	22.578
	7	-6	16.691	20.932
	8	-5.9	4.392	7.431
	9	-5.9	4.14	9.734
14	1	-6.7	0	0
	2	-6.6	2.927	7.831
	3	-5.9	20.856	23.422
	4	-5.8	3.917	5.436
	5	-5.7	4.942	7.578
	6	-5.7	10.073	13.779
	7	-5.6	4.018	6.215
	8	-5.6	8.963	12.268
	9	-5.6	8.734	12.287

@ Binding energies between ligand and receptor (Affinity (kcal/mol)).

# RMSD L.B: Distance from best mode root-mean-square deviation lower bound.

* RMSD U.B: Distance from best mode root-mean-square deviation upper bound.
